# The *PHEX* deletion variant (p.Thr605MetfsTer14) causes X-linked hypophosphatemic rickets by reducing protein expression and promoting mineralization

**DOI:** 10.1186/s13023-026-04354-x

**Published:** 2026-04-18

**Authors:** Zhongzhi Gan, Meiyu Zheng, Chenzhao Guo, Fei He, Yingchun Zheng, Jiayi Li, Chen Li, Fang Yang, Qingfang Song, Qingxian Chang, Qian Liu, Fu Xiong, Hairui Xie

**Affiliations:** 1 Department of Medical Genetics/Experimental Education/Administration Center, School of Basic Medical Sciences, Southern Medical University, Guangzhou, 510515 China; 2 Department of Pediatric Endocrinology and Well Child Care, Zhujiang Hospital, Southern Medical University, Guangzhou, 510280 China; 3 The First Clinical Medical School of Nanfang Hospital, Southern Medical University, Guangzhou, 510515 China; 4 Department of Fetal Medicine and Prenatal Diagnosis, Zhujiang Hospital, Southern Medical University, Guangzhou, 510280 China; 5 Shenzhen Maternity and Child Healthcare Hospital, Shenzhen Key Laboratory of Birth Defect Prevention and Control, Southern Medical University, Shenzhen, 518100 China

**Keywords:** XLH, *PHEX*, p.Thr605MetfsTer14, Protein stability, Mineralization

## Abstract

**Background:**

X-linked hypophosphatemic rickets (XLH, OMIM: 307800) is the most prevalent hereditary ricket characterized by hypophosphatemia, rickets, and osteomalacia. XLH resulted from pathogenic variants in *PHEX* gene. Patients with XLH may experience symptoms that begin in childhood and persist into adulthood or even throughout life, significantly impacting their quality of life. Therefore, studying the genetic etiology and pathogenic mechanisms of XLH is fundamental for the prevention, diagnosis, and treatment of the disease.

**Results:**

We identified a novel pathogenic variant in *PHEX* within XLH families: c.1814delC p.Thr605MetfsTer14. This variant has not been previously reported in patients with XLH. In in vitro functional studies, the variant (c.1814delC p.Thr605MetfsTer14) reduced the protein expression and stability of PHEX and altered its localization. Furthermore, the overexpression of wild-type PHEX inhibited mineralization in contrast to the control group. The PHEX variant (p.Thr605MetfsTer14) exhibited a greater capacity to promote mineralization than the wild-type group.

**Conclusions:**

We propose a potential correlation between reduced protein degradation rate and XLH phenotypes. The PHEX variant protein (p.Thr605MetfsTer14) changed the protein structure, induced mislocalization, accelerated protein degradation, and finally caused a decrease in protein expression, which affected mineralization. Our findings provide valuable data on XLH pathogenesis and genotype-phenotype correlations. The expression and mineralization studies of the PHEX variant offer important insights into the pathogenesis of XLH disease.

## Introduction

X-linked hypophosphatemic rickets (XLH, OMIM: 307800) is a hereditary disease marked by hypophosphatemia, rickets, and osteomalacia [1]. XLH results from pathogenic variants in the *PHEX* gene. With an estimated prevalence of 1/20,000, XLH is the most prevalent hereditary rickets [2, 3]. Children with XLH exhibit hypophosphatemia, rickets, and significantly impaired tooth and bone mineralization [3]. As they age, adult patients may develop complications such as early-onset osteoarthritis, enthesopathies, spinal stenosis, pseudo-fractures, and hearing loss [4]. The primary biochemical features of XLH include hypophosphatemia, elevated circulating fibroblast growth factor 23 (FGF23) concentrations, low or normal levels of 1.25(OH)2D or calcitriol, normal or slightly increased serum PTH, and elevated levels of serum alkaline phosphatase [5]. Recent research has found that the symptoms experienced by patients with XLH in childhood often persist into adulthood, resulting in a lower quality of life compared to the general population [6]. Although optimal treatment methods for XLH exist, achieving complete phenotypic rescue is rare [7]. Therefore, it is of great significance to investigate the genetic etiology and molecular pathophysiology of *PHEX*-related XLH for its preventive measures, diagnosis, and treatment.

The *PHEX* gene, located on the X chromosome, consists of 22 exons and encodes a transmembrane protein of 749 amino acids, with intracellular, transmembrane, and extracellular domains [8]. PHEX belongs to the zinc metallopeptidase family and is expressed in specific tissues, including healthy dento-osseous lineage cells, such as osteocytes, osteoblasts, and odontoblasts [9, 10]. Through the PHEX-ASARM interaction, PHEX inhibits the production of FGF23 [11, 12]. Subsequently, FGF23 upregulates phosphate excretion by downregulating the sodium phosphate (NaPi) transporter protein in the kidneys [13]. In conjunction with osteoblasts, osteoclasts, and extracellular matrix proteins, calcium and phosphate metabolism in the skeleton facilitates the osteoid mineralization during deposition [14]. The currently accepted mechanism of XLH is that PHEX inactivation results in excess FGF23, which reduces renal phosphorus reabsorption and consequently affects bone mineralization [7, 15]. According to the *PHEX* LSDB database (https://www.rarediseasegenes.com/), more than 800 pathogenic variants of the *PHEX* gene, which can occur at any position in the gene and include missense variants, nonsense, copy-number, frameshift and splicing sites, have been identified in patients with XLH [16, 17]. More than 70% of these variants result in truncated PHEX proteins [16]. Some studies have shown that patients with XLH with truncated variants tend to develop more severe bone diseases [18]. The exact molecular mechanisms *l*eading to the imbalance in phosphate metabolism and XLH remain unclear, despite the discovery of numerous XLH-associated variants in PHEX [19].

In this study, we identified a case of XLH caused by a novel heterozygous deletion variant (c.1814delC, p.Thr605MetfsTer14) in *PHEX*. The possible pathogenesis of XLH was further explored in this case. We investigated the pathogenesis of XLH by analyzing the protein structure, expression, stability, localization, and cellular mineralization of the mutated PHEX protein. Ultimately, these in vitro experimental studies further elucidate the abnormal mineralization seen in XLH.

## Materials and methods

### Patients and clinical examination

This study was performed in accordance with the ethical committee standards at the Zhujiang Hospital of Southern Medical University, China. We investigated a patient with hypophosphatemic rickets. Skilled clinical doctors gathered patient medical information, medication history, and family medical history. Peripheral venous blood was obtained from the parents (I:1 and I:2) and patient (II:1) with XLH. The patient underwent a physical examination, blood and urine biochemistry, and an X-ray. Informed written consent was received from the patient’s parents before conducting the molecular studies for this project.

### Variant analysis

Blood samples from peripheral veins were obtained and sent for genetic testing. Based on detailed family information, the pedigree was drawn. We performed whole-exome sequencing (WES) using genomic DNA for parents I:1 and I:2 and patient II:1. The WES was conducted by Guangzhou Jiajian Medical Testing Company (Guangzhou, China), resulting in the discovery of a significant variant in the *PHEX* gene.

### Bioinformatics analysis

I-TASSER and Pymol were used to estimate the three-dimensional (3D) protein structures.The harmfulness and pathogenicity of the PHEX were analyzed using Mutation Taster. Furthermore, ClusterX software was used to align multiple *PHEX* sequences from different species obtained from the Uniprot database.

### Plasmid construction and mutagenesis

Peripheral blood lymphocytes were treated with Trizol reagent (Invitrogen, Carlsbad, CA, USA) to extract total RNA. Following the directions, first-strand cDNA was created using the HiScript II 1st Strand cDNA Synthesis Kit (Vazyme, Jiangsu, China). According to the manufacturer’s instructions, the coding sequence (CDS) region of *PHEX* was amplified using the Phanta Max Super-Fidelity DNA Polymerase (Vazyme, Jiangsu, China). The primers were as follows: forward primer: 5′-ATGGAAGCAGAAACAGGGAGC‐3′; reverse primer: 5′‐CTACCAGAGTCGGCAGGAGT‐3′. Then, the products were cloned, respectively, into the *pEGFP-C1* and *pLVX-Mcherry-C1* vectors. After being purified, the plasmid was transformed into DH5α competent Escherichia coli (Vazyme, Jiangsu, China), and after antibiotic screening and sequencing, *pEGFP-PHEX-WT* and *pLVX-Mcherry-PHEX-WT* plasmids were obtained. In addition, mutant plasmids were generated by PCR-based site-specific mutagenesis, and the primers were as follows: forward primer: 5′‐GATCCTTGGTGGTCTATGAATCAGAAGAAAA‐3′; reverse primer: 5′‐TTTTCTTCTGATTCATAGACCACCAAGGATC‐3′. The plasmids of wild-type (*pEGFP-PHEX-WT* and *pLVX-Mcherry-WT*) or mutant *PHEX* genes (*pEGFP-PHEX-MUT* and *pLVX-Mcherry-MUT*) were obtained. Recombinant plasmids were extracted and purified using the TIANprep Mini Plasmid Kit (TIANGEN, Beijing, China).

### Virus packaging and cell infection

When human embryonic kidney (HEK) 293T cells were cultivated to a confluence of 75%, PEI was used to transfect the following lentivirus vectors: *pLVX-Mcherry-C1*, *pLVX-Mcherry-PHEX-WT*, *pLVX-Mcherry-PHEX-MUT*, *PSPAX2*, and *PMD2G* according to the instructions. A 0.45 μm filter was used to collect and filter the cell supernatant after 72 h. Then lentivirus was obtained by high-speed centrifugation. Ultracentrifugation was performed for two hours at 4 °C and 20,000 rpm to obtain the viral suspension.

The lentivirus was used to infect well-grown MG63 and 143B cells. After screening for 1–10 ug/mL puromycin for 7 days, Western Blot, qPCR, and PCR amplification were used to determine the overexpression PHEX stable transgenic cell lines.

### Western blotting analysis

When human embryonic kidney (HEK) 293T cells grew to a confluence of 75%, Lipofectamine 2000 (Invitrogen) was utilized for the transfection of recombinant vectors *pEGFP-C1*, *pEGFP-PHEX-WT*, and *pEGFP-PHEX-MUT*. The cells were collected forty-eight hours after transfection. Cell lysis buffer (Beyotime Biotechnology, Shanghai, China) supplemented with 1% phenylmethylsulfonyl fluoride (PMSF, Beyotime Biotechnology) was used to lyse the cells and prevent protein degradation. After the removal of cell debris by centrifugation, 5× loading buffer was added and mixed evenly, and then heat denaturation was carried out by heating at 100℃ for ten minutes. The obtained protein samples were separated by SDS-PAGE and transferred to a PVDF membrane (Millipore, MA, USA). Following a 90-minute incubation at room temperature with 5% nonfat milk, the membranes were treated overnight at 4 °C with either GFP-tagged mouse monoclonal antibodies (Proteintech, Rosemont, USA) or anti-β-Tubulin rabbit monoclonal antibodies (Proteintech, Rosemont, USA). The next day, after washing three times with TBST, the membranes were incubated with goat anti-mouse IgG or anti-rabbit IgG at room temperature for 90 min. SuperSignal West Pico ECL (Thermo Fisher Scientific) and a digital chemiluminescence system (Tanon Science & Technology, Shanghai, China) were used to detect protein expression.

Protein extraction and western blot of MG63 and 143B cells were the same as HEK 293T. Primary antibodies were Mcherry-tagged antibody and β-Tubulin antibody (Proteintech, Rosemont, USA). ImageJ software was used to quantify the protein band intensities. PHEX protein was normalized to β-tubulin.

### Protein degradation rate analyses

To determine if different genetic variants affected the protein degradation properties of PHEX, HEK293T cells were positioned in a 12-well dish and transfected using the *pEGFP-PHEX-WT* or *pEGFP-PHEX-MUT* plasmids. Then, at 36 h post-transfection, cycloheximide (CHX, Sigma-Aldrich) 100 µg/mL treated HEK293T cells. Total cellular protein was extracted at 0, 3, 6, and 9 h in order to assess the half-life of PHEX using western blotting.

### RNA analysis

Total RNA from transfected 293T and virus-infected 143B cells was obtained using TRIzol reagent. A total of 1 µg RNA was reverse-transcribed into cDNA using HiScript II Q RT SuperMix for qPCR (+ gDNA wiper) (Vazyme, Jiangsu, China). PCR was performed with ChamQ SYBR Color qPCR Master Mix (Vazyme, Jiangsu, China) to measure the relative mRNA levels of PHEX, *RUNX2*, *ALP*, and *OPN* genes. Primers for qPCR were as follows: *GAPDH*: F 5′‐GTGAAGGTCGGAGTCAACG‐3′ and R 5′‐TGAGGTCAATGAAGGGGTC‐3′; *PHEX*: F 5′‐CATCAGACCATCTACAGTGCCC‐3′′ and R 5′‐ACGTCGTACCCGTTTTCCAG‐3′; *RUNX2*: F 5′‐GAACCCAGAAGGCACAGACA‐3′ and R 5′‐GGCTCAGGTAGGAGGGGTAA‐3′; *ALP*: F 5′‐ACTGGGGCCTGAGATACCC‐3′ and R 5′‐TCGTGTTGCACTGGTTAAAGC‐3′; *OPN*: F 5′‐CTCCATTGACTCGAACGACTC‐3′ and R 5′‐CAGGTCTGCGAAACTTCTTAGAT‐3′. The transfection and real-time PCR experiments were conducted three times to verify the results’ repeatability.

### Cellular localization and semi-quantitative fluorescence

When the 293T cells reached 60%–70% confluence, the plasmids *pEGFP-C1* or *pEGFP-PHEX-WT* or *pEGFP-PHEX-MUT* were transfected into the cells by Lipofectamine 2000 (Invitrogen). The cells were treated with 4% paraformaldehyde and 0.2% Triton X-100 after forty-eight hours of transfection. Finally, the cells were incubated with DAPI for 15–20 min and viewed with a confocal fluorescence microscope (LSM 880; Carl Zeiss AG, Jena, Germany).

The stable 143B cell line and stable MG63 cell line of PHEX overexpression were inoculated on confocal dish 1 day in advance. The sample preparation for confocal fluorescence microscopy was the same as the 293T mentioned above. In addition, under the same red fluorescence parameter settings, the expression of PHEX fluorescence in the 143B stable transgenic cell line was semi-quantitatively analyzed using Nikon fluorescence microscopy (ECLIPSE Ti2; Nikon; Japan) and ImageJ software.

### Mineralization assays

The virus-infected stable MG63 or 143B cells in good growth conditions were sown in 12-well cell plates after cell counting. The solution was replaced with osteogenic induction media when the cell confluence reached about 75%. After 14–21 days of induction, the media was discarded, fixed with 4% paraformaldehyde, and dyed with alizarin red (LEAGENE, Beijing, China). After rinsing with PBS buffer for three times, the mineralized nodules were observed under a microscope (ECLIPSE Ti2; Nikon; Japan) and photographed. Next, the expression of *RUNX2*, *ALP*, and *OPN* in mineralized 143B cells was examined by RT-QPCR.

### Statistical analyses

GraphPad Prism software was used for the statistical analyses. Independent samples t-test and two-way ANOVA were used to determine the statistical significance of differences between groups. Statistical data are expressed as the mean ± standard deviation (SD). A P value < 0.05 was considered statistically significant.

## Result

### Clinical phenotype

**Fig. 1:**
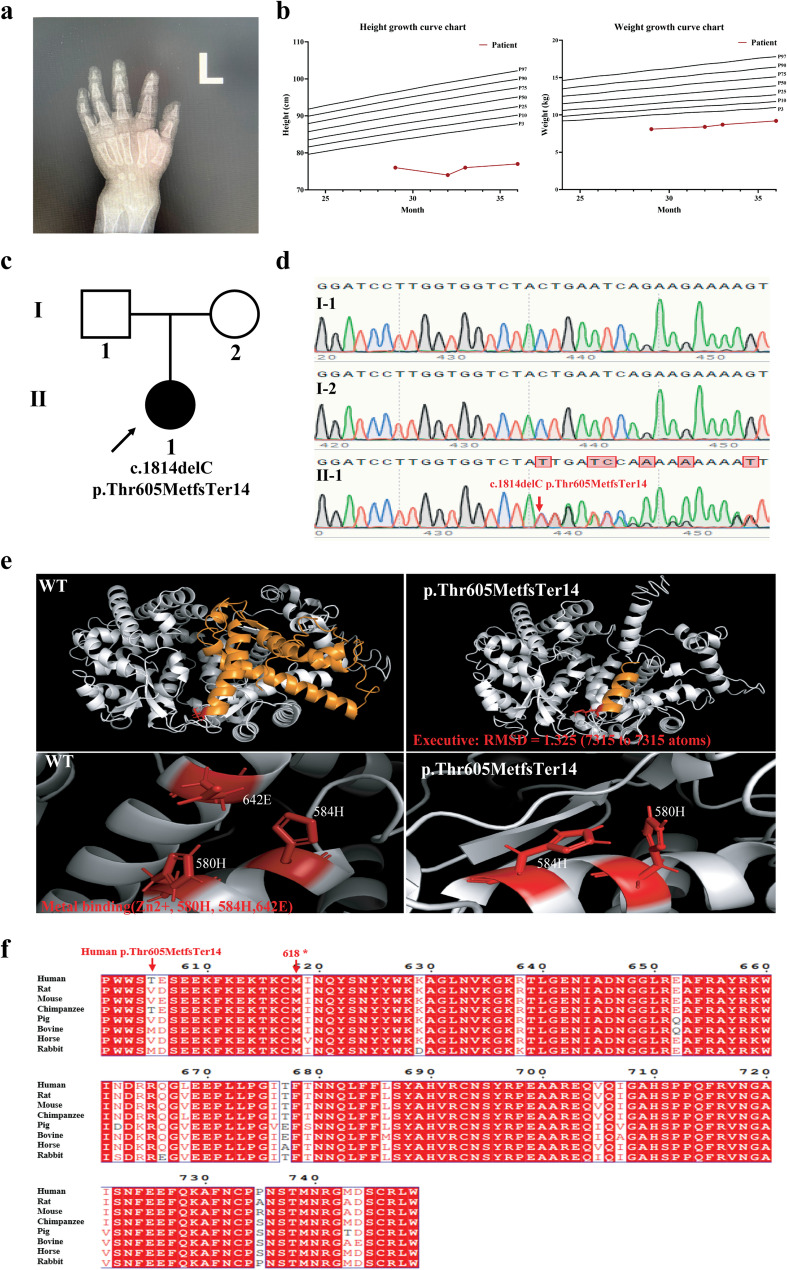
Analysis of clinical phenotype, variant and protein structure.** a** X-ray of the distal end of the carpal bones. **b** Height and weight growth curve chart. The red lines represent the patient’s height and weight indicators. The black line represents the height and weight of children aged 2–3, according to WHO statistics. P represents the percentile. For example, P3 represents 3% of the population in the same age group. **c** Family pedigree. Females are represented by circles, and males by squares. The proband is indicated with an arrow. The filled-black symbols indicate affected individuals. **d** DNA Sanger sequencing results. **e** Protein structure was analyzed by I-TASSER. The Zn^2+^ active region includes the sites 580H, 584H and 642E. **f** Amino acid sequence alignment of PHEX orthologues across different species

A 2.5-year-old girl from China was taken to Zhujiang Hospital of Southern Medical University due to gait instability and growth retardation. She was diagnosed with short stature and hypophosphatemic rickets. Growth retardation had been observed since she was 1.5 years old. An X-ray showed a moth-eaten-like change in the distal end of the carpal bones (Fig. 1a). The patient’s height and weight are below the 3rd percentile (P3) for the same age group, indicating severe underdevelopment (Fig. 1b). Biochemical examination showed decreased levels of serum inorganic phosphorus, whole blood calcium, urine calcium and phosphorus, while serum alkaline phosphatase levels were increased (Table 1). The bone mineral density level was − 5 SD. Physical examination revealed a bracelet anklet sign, pectus carinatum, and eversion of the costal margin. Her parents had no corresponding clinical manifestations.

**Table 1:** Clinical features of the patient

Age (year, month, day)	Sample	Clinical examination	
2Y5M29D	serum	Alkaline phosphatase (ALP) 117–390 IU/L	↑ 1080 IU/L
2Y5M29D	serum	Inorganic Phosphorus(*P*) 0.90–1.70 mmol/L	↓ 0.56 mmol/L
2Y5M29D	whole blood	Trace element calcium (Ca_A) 1.57–2.174 mmol/L	↓ 1.5 mmol/L
2Y8M3D	24-hour urine	Trace element calcium_U24h 2.5–7.5 mmol/L	↓ 0.1 mmoL/24 h
2Y8M3D	24-hour urine	Phosphorus_U24h 22.6–48.4 mmol/L	↓ 3.9 mmoL/24 h
3Y	24-hour urine	Trace element calcium_U24h 2.5–7.5 mmol/L	↓ 0.6 mmoL/24 h
3Y	24-hour urine	Phosphorus_U24h 22.6–48.4 mmol/L	↓ 2.8 mmoL/24 h
3Y2D	serum	Inorganic Phosphorus(P) 0.90–1.70 mmol/L	↓ 0.51 mmol/L
3Y2D	serum	Alkaline phosphatase (ALP) 117–390 IU/L	↑ 925 IU/L

### Variant analysis and bioinformatics analysis

The whole blood DNA of the parents and patient was delivered to Guangzhou Jiajian Medical Testing Corporation. Sequencing revealed a heterozygous variant c.1814del (p.Thr605MetfsTer14) of the *PHEX* gene in patient Ⅱ-1 (Fig. 1c-d). Sequencing data showed that her parents (Ⅰ-1 and Ⅰ-2) did not carry this variant (Fig. 1c-d). This variant is a frameshift variant predicted to cause premature stop codes in protein synthesis. The prediction results from Mutation Taster indicated that the c.1814del (p.Thr605MetfsTer14) variant in the *PHEX* gene is disease-causing and may act through five possible pathogenic mechanisms: (1) nonsense-mediated decay (NMD), (2) amino acid sequence change, (3) frameshift, (4) affecting protein features, and (5) splice site change. The *PHEX* variant (c.1814del, p.Thr605MetfsTer14) is a novel variant that has not been reported in the literature or the gnomAD database. We predicted the 3D structure of PHEX wild-type and variant proteins. The results showed that the variant produced a truncated protein, altering the tertiary structure and affecting the Zn^2+^ active region (Fig. 1e). Additionally, conservation analysis of the mutant sequence showed that most of the amino acids in the truncated part are highly conserved among species such as rats, mice, chimpanzees, bovines, horses, and rabbits (Fig. 1f). In summary, the bioinformatics prediction results indicate that the c.1814del (p.Thr605MetfsTer14) variant is pathogenic and harmful, affecting the Zn^2+^ active region.

### Expression analysis of PHEX variant

**Fig. 2:**
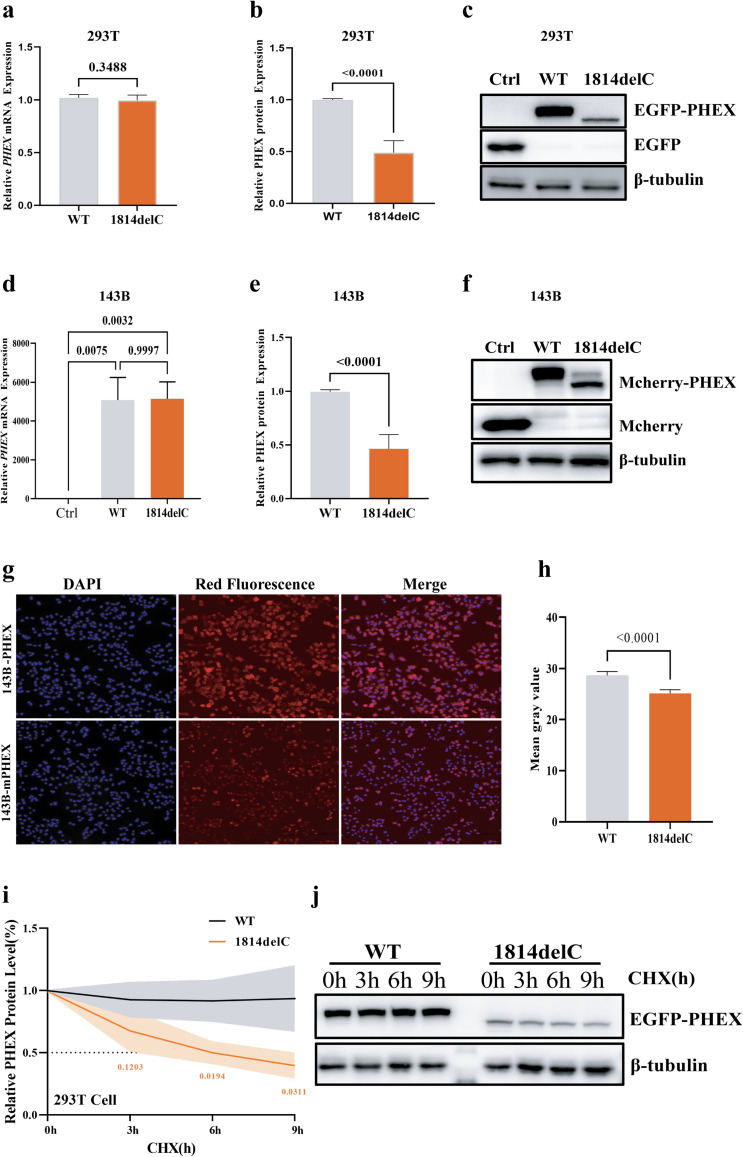
The effect of the variant c.1814del **(**p.Thr605MetfsTer14**) **on PHEX expression and protein degradation. **a** The mRNA expression levels of *PHEX* in 293T cells. The mRNA levels of the wild-type and variant did not change significantly (*p* > 0.05). **b-c** Western blotting analysis of PHEX in 293T cells. The expression level of the PHEX variant (c.1814delC) was lower than the wild-type (*p* < 0.0001). **d** The mRNA expression levels of *PHEX* in 143B cells. The mRNA levels of the wild-type and variant did not change significantly (*p* > 0.05). **e-f** Western blotting analysis of PHEX expression in 143B cells. The expression level of the PHEX variant (c.1814delC) was lower than the wild-type (*p* < 0.0001). **g-h** Semi-quantitative immunofluorescence analysis. Under the same red fluorescence parameter settings, the average fluorescence density of the 143B-mPHEX cell was lower than the wild-type 143B-PHEX cell. Red fluorescence represents the Mcherry-PHEX fusion protein. Object lens up to 10 magnifications. **i-j** PHEX protein degradation analyses. The variant protein (c. 1814delC, p.Thr605MetfsTer14) exhibited a shorter half-life in comparison to the wild-type

The expression of the *PHEX* variant was analyzed in HEK293T, 143B and MG63 cells. In 293T cells, we transiently transfected *pEGFP-C1*, *pEGFP-PHEX-WT* and *pEGFP-PHEX-MUT* plasmids. In these transiently overexpressed 293T cell lines, the expression of *PHEX* variant (c.1814del, p.Thr605MetfsTer14) showed no difference at the RNA level but was decreased at the protein level (Fig. 2a-c). To further study the effects of PHEX in 143B and MG63 cells, lentiviruses were packaged, including empty *pLVX-Mcherry-C1* plasmids, overexpressed wild-type *pLVX-Mcherry-WT* and mutant *pLVX-Mcherry-MUT* plasmids, and then successfully infected 143B and MG63 cells. After puromycin screening, we acquired the following six cell lines: 143B-pLVX, 143B-PHEX, 143B-mPHEX, MG63-pLVX, MG63-PHEX and MG63-mPHEX. The 143B-pLVX and MG63-pLVX served as control groups, 143B-PHEX and MG63-PHEX as wild-type groups, and 143B-mPHEX and MG63-mPHEX as mutant c.1814delC groups. In stable lentivirus-overexpressing 143B cells, the expression of PHEX followed the same trend as observed in transient transfection of 293T at both RNA and protein levels (Fig. 2d-f). Additionally, under the same red fluorescence parameter settings, we observed that the average fluorescence density of the 143B-mPHEX cell line was lower than that of the wild-type 143B-PHEX cell, indicating that the expression of PHEX variant (c.1814del, p.Thr605MetfsTer14) protein was lower than that in the wild-type group (Fig. 2g-h). Moreover, the PHEX variant protein also exhibited a shorter half-life in comparison with the wild-type 293T cells, indicating decreased stability (Fig. 2i–j).

### Cellular localization

**Fig. 3:**
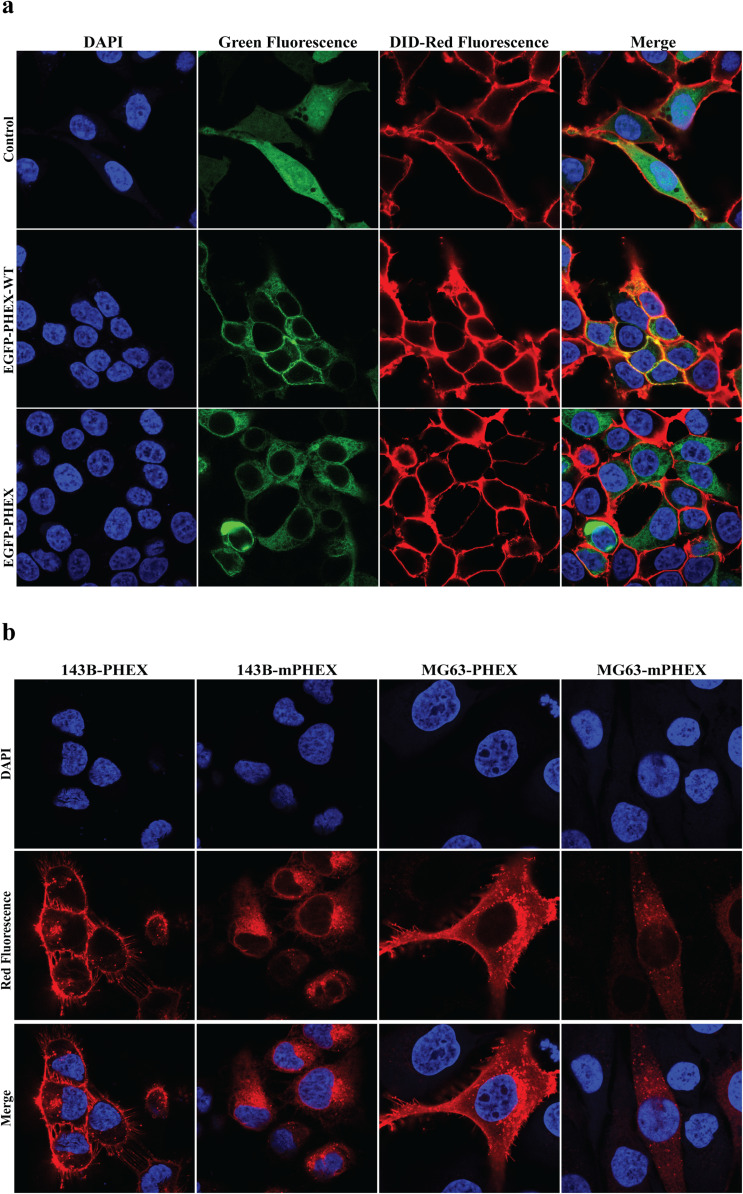
The subcellular localization of PHEX protein in 293T, 143B and MG63 cells.** a** Subcellular localization of PHEX in 293T cells. Confocal images of EGFP (green), DID (red), DAPI nuclear staining (blue), and merged signals. Green fluorescence represents the EGFP-PHEX fusion protein. Red fluorescence represents the cell membrane stained with DID. Object lens up to 100 magnifications. **b** Subcellular localization of PHEX in 143B and MG63 cells. Confocal images show Mcherry (red), DAPI nuclear staining (blue), and merged signals. Red fluorescence represents the Mcherry-PHEX fusion protein. Object lens up to 100 magnifications

The cellular localization of the PHEX variant was observed in HEK293T, 143B, and MG63 cells. We noticed differences in the subcellular localization of the PHEX variant (c.1814del, p.Thr605MetfsTer14) compared to the wild-type protein. The PHEX variant (c.1814del, p.Thr605MetfsTer14) was primarily localized in the cytoplasm, while the wild-type PHEX was found in both the cell membrane and cytoplasm (Fig. 3a-b).

### Mineralization induction of 143B and MG63

**Fig. 4:**
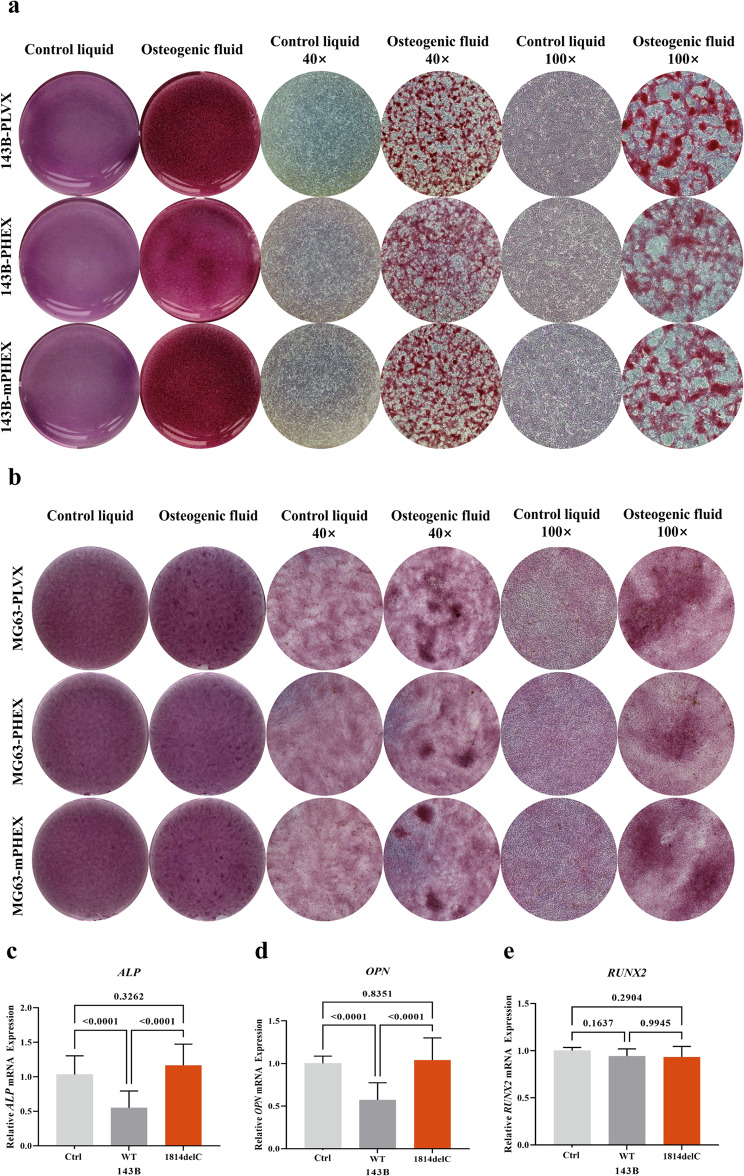
Mineralization induction of 143B and MG63.** a** Representative image from the ARS staining of 143B-PLVX, 143B-PHEX and 143B-mPHEX cells after differentiation induction. Object lens up to 4 and 10 magnifications. **b** Representative images from the ARS staining of MG63-PLVX, MG63-PHEX and MG63-mPHEX cells after differentiation induction. Object lens up to 4 and 10 magnifications. **c-e** The expression levels of *ALP*, *OPN* and *RUNX2* mRNA in 143B cells at 14 days after differentiation. The expression of *ALP* and *OPN* in the control and mutation group (1814delC) was significantly higher than that in the wild-type group at 14 days after differentiation

To investigate if the mutated PHEX affects the function of 143B and MG63 cells, we performed Alizarin Red S (ARS) staining on cells incubated in osteogenic induction medium for 14 days to examine mineralized nodule deposition. After 14 days of osteogenic induction in 143B and MG63 cells, wild-type PHEX overexpression decreased mineralized nodule deposition compared to the control groups (143B-pLVX and MG63-pLVX), while the PHEX variant showed an increased capacity to induce mineralization in comparison with the wild-type PHEX (Fig. 4a-b). Compared to the wild type, the number of mineralized nodules in the control and mutation groups was higher, the volume of mineralized nodules was larger, and the staining was more intense. To further determine whether the PHEX variant (p.Thr605MetfsTer14) affects the mineralization induction ability of 143B cells, we analyzed the expression of *RUNX2*, *ALP*, and *OPN* mRNA after mineralization induction by RT-qPCR. The expression of *ALP* and *OPN* in the control and mutation groups was significantly higher than that in the wild-type group 14 days after differentiation (Fig. 4c-d). However, there was no significant difference in *RUNX2* between the three groups (Fig. 4e). In summary, compared to 143B-PLVX and MG63-PLVX, the overexpression of wild-type PHEX inhibited mineralization. Compared to 143B-PHEX and MG63-PHEX, the variant (p.Thr605MetfsTer14) had a higher ability to promote mineralization.

## Discussion

XLH is primarily caused by a deficiency of PHEX, leading to an increase in FGF23. FGF23 acts on the kidneys to inhibit the production of 1,25-dihydroxyvitamin D and reduce phosphate reabsorption, resulting in hypophosphatemia, which leads to poor mineralization of the extracellular matrix in the bones and teeth of patients with XLH [20]. In this study, a 2.5-year-old child was diagnosed with X-linked hypophosphatemic rickets due to a novel *PHEX* heterozygous variant (c. 1814delC, p.Thr605MetfsTer14), based on genetic and phenotype analysis. Diagnosis is frequently delayed due to the rarity of XLH and the variety of clinical symptoms [4]. Therefore, studying the molecular function of PHEX is helpful to understand the XLH disease, improve the diagnosis and provide effective treatment.

According to the bioinformatics prediction results, the c.1814del (p.Thr605MetfsTer14) variant is a novel variant characterized by pathogenicity, harmfulness and conservation. Pathogenic variants in *PHEX* can significantly alter the protein’s location and structure, thereby restricting its functionality. For instance, the PHEX missense variants (p.G579R, p.C85R, and p.F727L) impair protein trafficking, confining the PHEX variant protein to the endoplasmic reticulum, preventing it from reaching the plasma membrane [21, 22]. The nonsense variant c.148 A > T in the transmembrane domain causes premature termination of the PHEX protein, which leads to the loss of the entire extracellular domain and destruction of the PHEX protein’s functionality [23]. Furthermore, more variants have been found in the C-terminal region of the PHEX protein than in the N-terminal region, and there are zinc binding sites at the C-terminus, the lack of which leads to the loss of PHEX activity [24]. Here, we discovered that the PHEX c.1814del variant causes a shortened protein, changes the active region of the zinc binding site, and prevents it from locating the plasma membrane. Because the variant protein (p.Thr605MetfsTer14) is unable to locate the plasma membrane, it fails to function normally. This finding further provides strong evidence supporting the pathogenicity of this variant locus (c.1814delC, p.Thr605MetfsTer14).

To further understand how the variant (c.1814delC, p.Thr605MetfsTer14) causes the disease, in vitro functional experiments were performed. Variants such as p.Trp749Arg and p.Glu145* have reportedly been shown to decrease PHEX levels [25]. According to researchers like Huang J, Bao X, and Xia W, the *PHEX* variant (c.1692 del A) causes the protein to lose both its putative active sites and its zinc binding sites, accelerating PHEX degradation [26]. In our study, we confirm that the PHEX variant protein (p.Thr605MetfsTer14) leads to the loss of zinc binding sites and putative active sites, ultimately causing protein localization errors, hastening protein degradation, and reducing protein expression. Additionally, one study found that mineralization defects in *Hyp* mice are not affected by gene dosage [27]. However, other researchers have observed that homozygous *phex* female mice are significantly smaller, have shorter femurs, and have lower bone density compared to heterozygous females, indicating a dose-response in the XLH skeletal phenotype [28]. We hypothesize that the degradation rate of the variant proteins may correlate with the severity of the illness. Further research is needed to verify whether the severity of the disease is influenced by the degradation rate of variant proteins.

On the other hand, in our study, the fact that the p.Thr605MetfsTer14 variant enhances mineralization appears to contradict the XLH condition of impaired bone mineralization. An in vitro study showed that overexpression of Phex was insufficient to rescue the mineralization phenotype of *Hyp* osteoblasts [29]. In addition, in transgenic mice (PHEX-tg) under the control of a human β-actin promoter, overexpression of PHEX did not lead to changes in mineral ion levels or bone homeostasis [30]. In our study, it was observed that overexpression of wild-type PHEX inhibited mineralization in MG63-PHEX and 143B-PHEX cells compared with MG63-PLVX and 143B-PLVX cells. Therefore, we believe that overexpression of PHEX cannot promote cell mineralization and may even prevent mineralization in some cases. Furthermore, contrary to the mineralization defects in XLH, PHEX-KO iPSCs accelerated mineralization following osteoinduction [31]. We also noticed a decrease in PHEX expression and accelerated mineralization in MG63-mPHEX and 143B-mPHEX compared to the wild group (143B-PHEX and MG63-PHEX). This may be related to the enthesopathy associated with abnormal mineralization in XLH. Enthesopathy, characterized by abnormal mineralization at the tendon-bone attachment site, is a significant complication in adults with XLH [32]. Thus, we believe that the formation of mineralized osteoblasts is impacted by the PHEX variant protein (p.Thr605MetfsTer14).

In conclusion, the PHEX variant protein (p.Thr605MetfsTer14) altered the protein structure, caused protein localization errors, accelerated protein degradation, and ultimately caused a decrease in protein expression, thereby accelerating mineralization. Stability and mineralization studies of the PHEX variant provide important insights into the pathogenesis of XLH disease. We provide useful data on XLH pathogenesis.

## Data Availability

The datasets used and/or analyzed during the current study are available from the corresponding author on reasonable request.
